# Targeting of Intracellular TMEM16 Proteins to the Plasma Membrane and Activation by Purinergic Signaling

**DOI:** 10.3390/ijms21114065

**Published:** 2020-06-05

**Authors:** Rainer Schreiber, Jiraporn Ousingsawat, Karl Kunzelmann

**Affiliations:** Institut für Physiologie, Universität Regensburg, Universitätsstr. 31, 93053 Regensburg, Germany; jiraporn.ousingsawat@ur.de (J.O.); karl.kunzelmann@ur.de (K.K.)

**Keywords:** ANO5, ANO8, ANO9, ANO10, TMEM16E, TMEM16H, TMEM16J, TMEM16K, scramblase, membrane blebbing, P2X7R, purineric signaling

## Abstract

Anoctamins such as TMEM16A and TMEM16B are Ca^2+^-dependent Cl^−^ channels activated through purinergic receptor signaling. TMEM16A (ANO1), TMEM16B (ANO2) and TMEM16F (ANO6) are predominantly expressed at the plasma membrane and are therefore well accessible for functional studies. While TMEM16A and TMEM16B form halide-selective ion channels, TMEM16F and probably TMEM16E operate as phospholipid scramblases and nonselective ion channels. Other TMEM16 paralogs are expressed mainly in intracellular compartments and are therefore difficult to study at the functional level. Here, we report that TMEM16E (ANO5), -H (ANO8), -J (ANO9) and K (ANO10) are targeted to the plasma membrane when fused to a C-terminal CAAX (cysteine, two aliphatic amino acids plus methionin, serine, alanin, cystein or glutamin) motif. These paralogs produce Ca^2+^-dependent ion channels. Surprisingly, expression of the TMEM16 paralogs in the plasma membrane did not produce additional scramblase activity. In contrast, endogenous scrambling induced by stimulation of purinergic P2X7 receptors was attenuated, in parallel with reduced plasma membrane blebbing. This could suggest that intracellular TMEM16 paralogs operate differently when compared to plasma membrane-localized TMEM16F, and may even stabilize intracellular membranes. Alternatively, CAAX tagging, which leads to expression in non-raft compartments of the plasma membrane, may antagonize phosphatidylserine exposure by endogenous raft-located TMEM16F. CAAX-containing constructs may be useful to further investigate the molecular properties of intracellular TMEM16 proteins.

## 1. Introduction

The TMEM16/Anoctamin family of proteins consists of ten transmembrane paralogs, ranging from TMEM16A/ANO1 to TMEM16K/ANO10. In addition to their function as Ca^2+^-activated Cl^−^ channels, some members of this family, like TMEM16F, also operate as Ca^2+^-activated lipid scramblases. Lipid scramblases facilitate the passive transport of phospholipids, such as phosphatidylserine (PtdSer), from the inner to the outer leaflet of the plasma membrane [1,2,3,4]. TMEM16A works exclusively as a Ca^2+^-activated Cl^−^ channel in secretory epithelial cells of the exocrine pancreas and salivary glands. TMEM16A is activated by extracellular ATP or UTP through stimulation of purinergic P2Y receptors and generation of inositol 1,4,5-trisphosphate (IP_3_) by phospholipase C, which leads to a Ca^2+^ release from endoplasmic reticulum (ER) stores [5]. Expression of TMEM16A also promotes cell proliferation and migration of tumor cells, and cyst growth in autosomal recessive polycystic kidney disease [6,7,8]. TMEM16E is required for skeletomuscular repair; its mutations cause gnathodiaphyseal dysplasia [9] and different types of muscular dystrophies [10]. Regarding TMEM16H and TMEM16J, there is very limited information available on their properties and cellular functions. For TMEM16H, Jha et al. reported its function as a key tether that forms ER/PM membrane contact sites to control intracellular Ca^2+^ signaling [11]. TMEM16J has a role in the progression and metastasis of colorectal cancer [12]. It is associated with poor prognosis of pancreatic cancer [13]. For TMEM16K, we reported a central role in the innate immune defense against Borrelia infection [14]. Moreover, mutations in TMEM16K cause recessive cerebellar ataxia [15], epilepsy, cognitive impairment [16], and coenzyme Q10 deficiency [17]. In contrast to TMEM16A, which is well expressed at the plasma membrane, TMEM16E, -H, -J and -K are predominantly expressed in membranes of intracellular compartments, in particular in the endoplasmic reticulum (ER) [4,11,14,18,19]. This makes it difficult to study the biophysical properties and mechanisms of activation of these TMEM16 paralogs. Here, we reported that TMEM16E, -H, -J and -K can be targeted to the plasma membrane by fusion with a C-terminal CAAX motif. Plasma membrane-targeted TMEM16 paralogs produced ion currents but did not scramble phospholipids. The results confirm an earlier report suggesting anoctamins as a family of Ca^2+^ activated ion channels [4]. CAAX-mediated plasma membrane targeting may be helpful to further investigate the molecular properties of intracellular TMEM16 proteins.

## 2. Results

### 2.1. Targeting TMEM16E, TMEM16H, TMEM16J and TMEM16K to the Plasma Membrane

**Figure 1:**
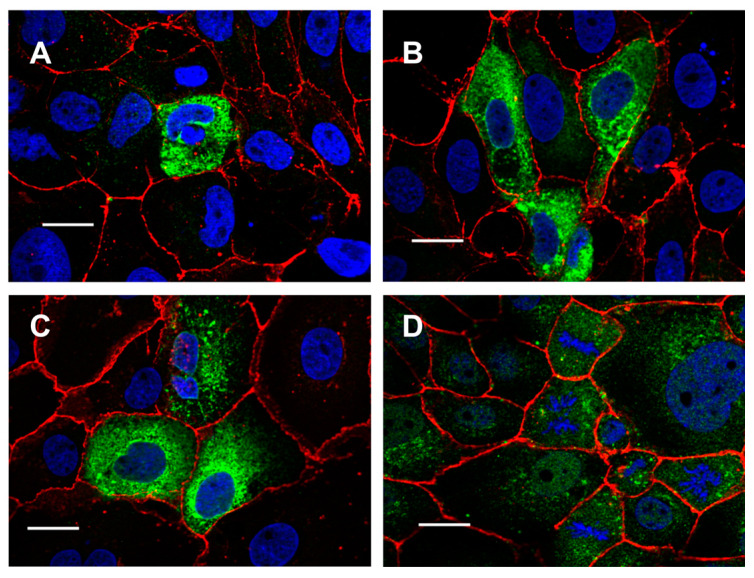
TMEM16E, -H, -J, and -K are expressed in intracellular compartments. Expression of (**A**) TMEM16E, (**B**) TMEM16H, (**C**) TMEM16J and (**D**) TMEM16K in FRT cells. His-tagged TMEM16E, -H, -J and -K proteins were detected with anti-His antibodies (green). β-catenin was visualized using an Alexa 568-labeled secondary antibody (red). Bar 20 µm.

**Figure 2:**
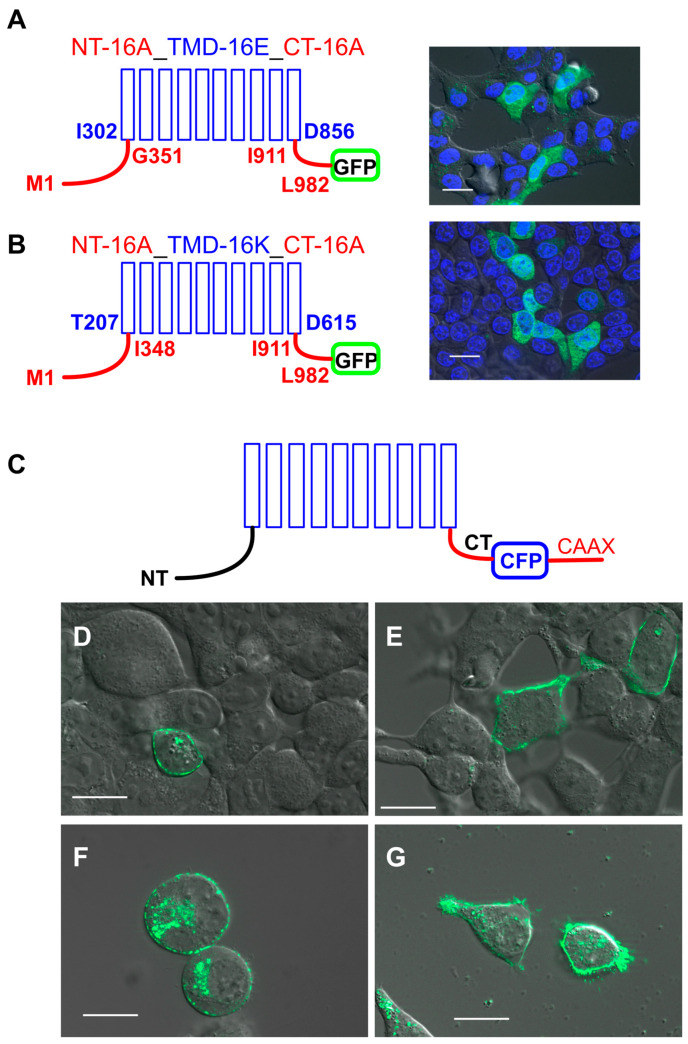
Expression of TMEM16 proteins. Chimeras of TMEM16A/TMEM16E and TMEM16A/TMEM16K were located in intracellular compartments (A,B), while TMEM16 paralogs were targeted by the CAAX motif to the plasma membrane (C-G). N-terminus (NT, amino acid (aa) M1-G351 or M1-I348) and C-terminus (CT, aa I911-L982) of TMEM16A (red lines) were cloned to the transmembrane domains (blue columns) of (**A**) TMEM16E (aa I302-D856) and (**B**) TMEM16K (aa T207-D615). In living HEK293 cells, localization of the chimeras was visualized by C-terminal-tagged GFP proteins (green). (**C**) Cyan fluorescent protein (CFP) (blue) and the CAAX motif (red) were cloned to the C-terminus (CT) of TMEM16 proteins. In living cells (**D**) TMEM16E-CFP-CAAX, (**E**) TMEM16H-CFP-CAAX, (**F**) TMEM16J-CFP-CAAX and (**G**) TMEM16K-CFP-CAAX were located at the plasma membrane. N-terminus (NT), Bar 20 µm.

In Fisher rat thyroid (FRT) cells, overexpressed His-tagged TMEM16E, TMEM16H, TMEM16J and TMEM16K, were located in membranes of intracellular compartments (Figure 1). Two different strategies were used to target these TMEM16 paralogs to the plasma membrane. As a first strategy, we fused the cytosolic N- and C-terminal parts of TMEM16A to the transmembrane domains of TMEM16E and TMEM16K. To visualize expression of these chimeras in living human embryonic kidney (HEK293) cells, the constructs were tagged with green fluorescent protein (GFP). However, these chimeras were still expressed intracellularly (Figure 2A,B). As a second strategy, we added the cyan fluorescent protein (CFP) to the CAAX motif sequence (KKKKSKTKCVIM) from Rho GTPase and fused both to the carboxyl terminus of TMEM16E, TMEM16H, TMEM16J and TMEM16K. It was shown by Agarwal et al. [20] that the CAAX motif targets the cAMP sensitive biosensor Epac2 specifically to non-lipid raft domains of the plasma membrane. At the C-terminus, CAAX proteins contain a specific recognition site (CAAX motif/box) for prenylation, a post-translational modification process. Due to prenylation, 15-carbon farnesyl groups or 20 carbon isoprenoids are added to the cysteine residues of the CAAX box [21]. Indeed, this translational modification guided TMEM16E-CFP-CAAX, TMEM16H-CFP-CAAX, TMEM16J-CFP-CAAX and TMEM16K-CFP-CAAX to the plasma membrane (Figure 2C–G).

### 2.2. Purinergic Stimulation and Increase in Intracellular Ca^2+^ Concentration Induces Anion Permeability in Plasma Membrane Targeted TMEM16E, TMEM16H, TMEM16J or TMEM16K

**Figure 3:**
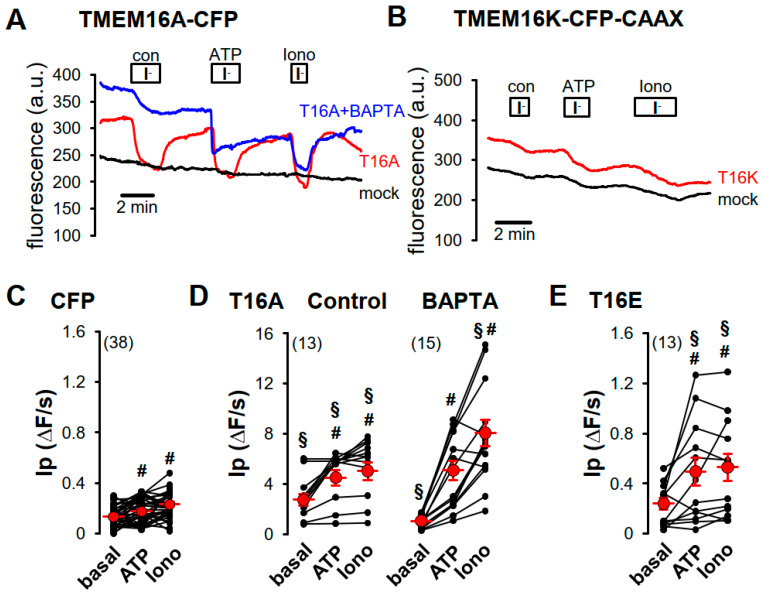

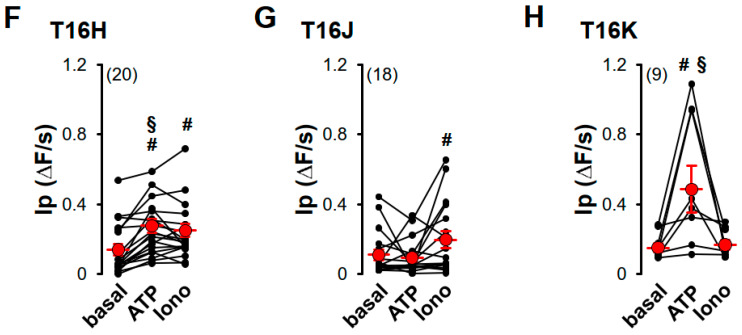
Purinergic stimulation and increase of intracellular Ca^2+^ concentration by ionomycin induced iodide permeability in HEK293-YFP cells expressing plasma membrane-targeted TMEM16 proteins. (**A**) Original recording of TMEM16A-CFP-expressing HEK293-YFP cells. Application of 20 mM iodide under control (con) in the presence of 100 µM ATP (ATP) or in the presence of 1 µM ionomycin (Iono) caused in TMEM16A-CFP-expressing HEK293-YFP cells (red, T16A) a strong but in TMEM16A-CFP-non-expressing HEK293-YFP cells (black, mock) only a weak transient quenching of the YFP signal, indicating enhanced iodide permeability through TMEM16A-CFP expression. The Ca^2+^-chelator BAPTA reduced iodide quenching under control conditions in TMEM16A-CFP-expressing HEK293-YFP cells (blue, T16A + BAPTA). (**B**) Original recording of TMEM16K-CFP-CAAX-expressing HEK293-YFP cells (red, T16K). Twenty mM iodide induced a slight increase of the YFP-quenching under control (con) and in the presence of 1 µM ionomycin (Iono), like in TMEM16K-CFP-CAAX-non-expressing HEK293-YFP cells (black, mock) but significantly increased YFP-quenching in the presence of 100 µM ATP when compared to control cells (mock). Summaries of iodide permeability (I_P_) measured by initial slope of fluorescence decrease (ΔF/s) of (**C**) CFP-expressing cells under basal condition (basal), in the presence of 100 µM ATP (ATP) or in the presence of 1 µM ionomycin (Iono); (**D**) of TMEM16A-CFP (T16A)-expressing HEK293-YFP cells under control condition or pre-incubated with 50 µM BAPTA for 30 min at RT; (**E**) TMEM16E-CFP-CAAX (T16E)-, (**F**) TMEM16H-CFP-CAAX (T16H)-, (**G**) TMEM16J-CFP-CAAX (T16J)-, or (**H**) TMEM16K-CFP-CAAX (T16K)-expressing HEK293-YFP cells. (Number of cells measured), # paired t-test, α < 0.05, § unpaired t-test to control and CFP, resepectively, α < 0.05, and analysis of variance (ANOVA) to CFP, α < 0.05.

**Figure 4:**
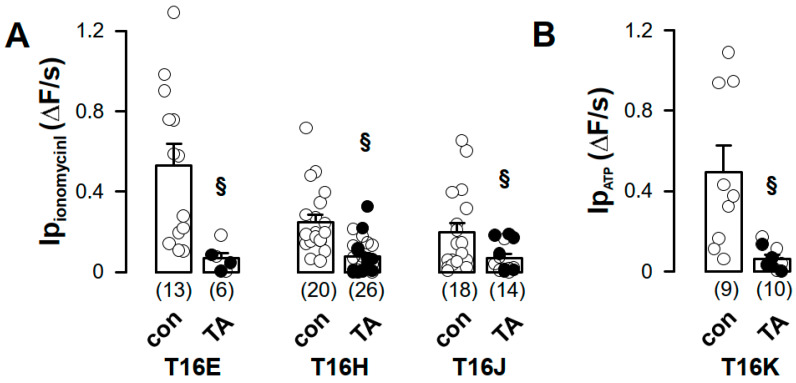
Increased iodide permeability induced by TMEM16 proteins was sensitive to tannic acid. (**A**) Ionomycin-induced iodide permeability (Ip_ionomycin_) from TMEM16E-CFP-CAAX (T16E)-, TMEM16H-CFP-CAAX (T16H)- or TMEM16J-CFP-CAAX (T16J)-expressing HEK293 cells was measured under control (con) or after pre-incubation with tannic acid (TA, 20 µM) for 3 min. (**B**) ATP-induced iodide permeability (Ip_ATP_) of TMEM16K-CFP-CAAX was measured under control (con) or after pre-incubation with tannic acid (TA, 20 µM) for 3 min. (Number of cells measured), § unpaired t-test, α < 0.05.

Anion permeability of TMEM16 proteins was determined in yellow fluorescence protein (YFP)-quenching assays using HEK293 cells stably expressing the iodide sensitive yellow fluorescent protein I152L (YFP) [22]. Validation of the YFP assay by the C-terminal CFP-tagged Ca^2+^-activated chloride channel TMEM16A (TMEM16A-CFP) showed fluorescence quenching after application of iodide (I^−^), upon stimulation with 100 µM ATP and in the presence of the Ca^2+^ ionophore ionomycin (Iono, 1 µM) (Figure 3A). The initial decrease of the YFP fluorescence by iodide (initial slope, ΔF/s) correlated to the iodide permeability (I_P_) and is summarized in Figure 3D. Compared to CFP-transfected cells (Figure 3C, mock), the basal iodide quenching was increased in TMEM16A-CFP-expressing cells, and was increased by ATP or ionomycin (Figure 3D). Pre-incubation of the cells for 30 min with the Ca^2+^-chelator BAPTA significantly decreased basal I_P_ and slightly increased the effect of ionomycin, due to increased sensitivity of unstimulated TMEM16A (Figure 3A,D). Expression of the TMEM16-CFP-CAAX proteins in HEK-YFP cells had no effect on basal I_P_. However, ATP or ionomycin induced in all TMEM16-CFP-CAAX proteins an increase of I_P_ (Figure 3B,E–H). For TMEM16E-CFP-CAAX and TMEM16H-CFP-CAAX, the ATP-induced increase of I_P_ was enhanced when compared to the CFP control (Figure 3C) but could not be further augmented by ionomycin (Figure 3E,F), whereas only ionomycin, but not ATP, increased I_P_ in TMEM16J-CFP-CAAX-expressing cells. This increase of I_P_ was not significantly different from the CFP control (Figure 3G). TMEM16K-CFP-CAAX-dependent I_P_ was only significantly activated by ATP when compared to CFP control (Figure 3B,H). Tannic acid (TA, 20 µM), a TMEM16 blocker [23], was used to confirm the activation of TMEM16 proteins. Indeed, ionomycin- or ATP-induced increases of I_P_ were sensitive to tannic acid (Figure 4A,B).

**Figure 5:**
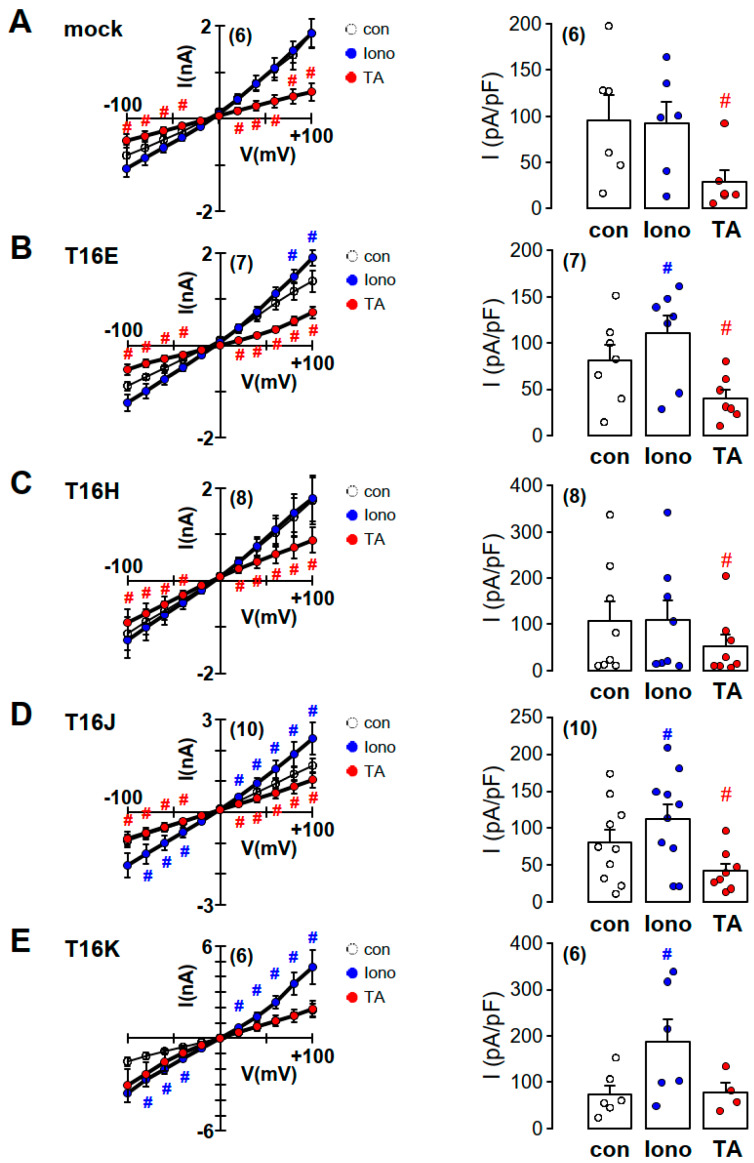
Increase of intracellular Ca^2+^ by ionomycin activated whole-cell Cl^−^ currents in TMEM16E-, J- and K-CFP-CAAX-expressing HEK293 cells. (**A**) Current/voltage relationships obtained in non-stimulated mock-transfected HEK293 cells (con), 1 µM ionomycin-treated cells (Iono) and ionomycin-treated cells pre-incubated with 20 µM tannic acid (TA). Summary of corresponding current densities obtained at Vc = +100 mV. (**B**) Current/voltage relationships obtained in TMEM16E-CFP-CAAX (T16E)-transfected HEK293 cells. Summary of corresponding current densities obtained at Vc = +100 mV. (**C**) Current/voltage relationships obtained in TMEM16H-CFP-CAAX (T16H)-transfected HEK293 cells. Summary of corresponding current densities obtained at Vc = +100 mV. (**D**) Current/voltage relationships obtained in TMEM16J-CFP-CAAX (T16J)-transfected HEK293 cells. Summary of corresponding current densities obtained at Vc = +100 mV. (**E**) Current/voltage relationships obtained in TMEM16K-CFP-CAAX (T16K)-transfected HEK293 cells. Summary of corresponding current densities obtained at Vc = +100 mV. (Number of cells measured), # paired t-test, α < 0.05.

These results were confirmed in additional whole-cell patch-clamp experiments (Figure 5). CFP-positive HEK293 cells were patched under whole-cell configuration with 145 mM CsCl_2_ and 10 nM Ca^2+^ as the pipette solution. Increase in intracellular Ca^2+^ concentration by 1 µM ionomycin induced in TMEM16E-, TMEM16J- and TMEM16K-CFP-CAAX-expressing HEK293 cells a rapid and transient whole-cell current which was sensitive to 20 µM tannic acid (Figure 5B,D,E). In mock-transfected and TMEM16H-CFP-CAAX-expressing HEK293 cells, ionomycin had no effect on whole-cell currents (Figure 5A,C).

### 2.3. Plasma Membrane-Targeted Expression of TMEM16E, TMEM16H, TMEM16J and TMEM16K Did Not Correlate to Phopholipid Scrambling Activity and Reduced P2X7R-Dependent Membrane Blebbing

**Figure 6:**
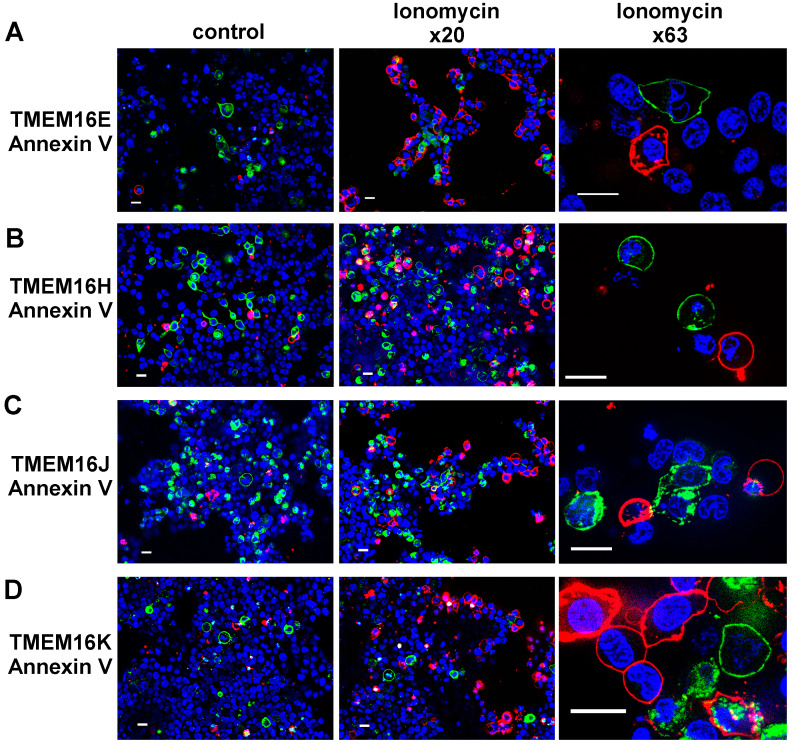

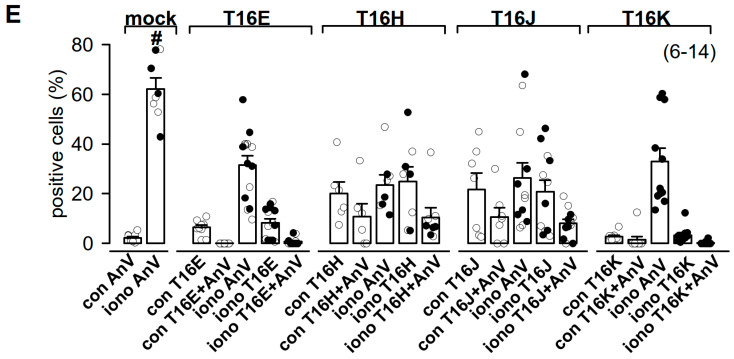
Plasma membrane expression of TMEM16E, -H, -J or -K was not correlated to phospholipid scrambling activity. (**A**) CFP fluorescence (green) of living HEK293 cells transfected with TMEM16E-CFP-CAAX, (**B**) TMEM16H-CFP-CAAX, (**C**) TMEM16J-CFP-CAAX or (**D**) TMEM16K-CFP-CAAX was mostly not localized with the annexin V signal (red), indicating absence of phosphatidylserine scrambling to the outer leaflet of the plasma membrane under control and after stimulation with ionomycin. Bar 20 µm. (**E**) Summary of positive cells for TMEM16 paralogs and annexin V staining under control (con) and stimulated (iono) conditions. (Number of experiments), # unpaired t-test, and analysis of variance (ANOVA), α < 0.05.

TMEM16E-CFP-CAAX proteins were only expressed in 6.4 ± 0.5 % of transfected HEK293 cells; TMEM16H-CFP-CAAX in 20 ± 4.8 %, TMEM16J-CFP-CAAX in 21.8 ± 6.6 % and TMEM16K-CFP-CAAX in 2.7 ± 0.5% (Figure 6E). Scrambling activity of living HEK293 cells was measured by detection of phosphatidylserine in the outer leaflet of the plasma membrane by Annexin V staining (Figure 6). Incubation of mock-transfected cells with 5 µM ionomycin for 10 min at RT resulted in an increased cell number positive for Annexin V from 2.2 ± 0.5% (con AnV) to 62.2 ± 4.4% (iono AnV, Figure 6E). In general, the ionomycin-induced scrambling was significantly reduced in cells transfected with TMEM16-CFP-CAAX paralogs (Figure 6E). In addition, ionomycin-induced scrambling activity was not correlated to the expression of TMEM16-CFP-CAAX paralogs. Only a fraction of TMEM16-CFP-CAAX paralogs-expressing cells showed scrambling activity under control conditions, which was not further enhanced by ionomycin (Figure 6).

**Figure 7:**
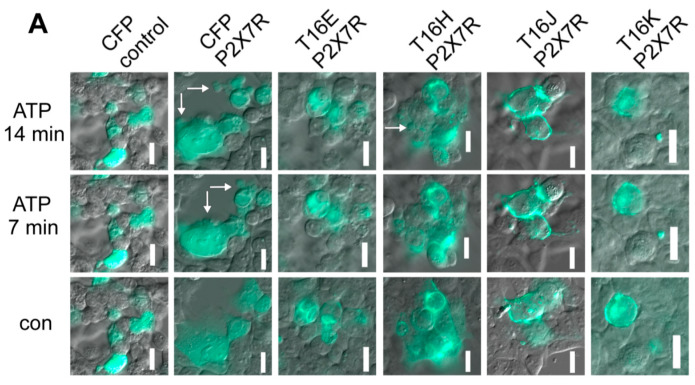

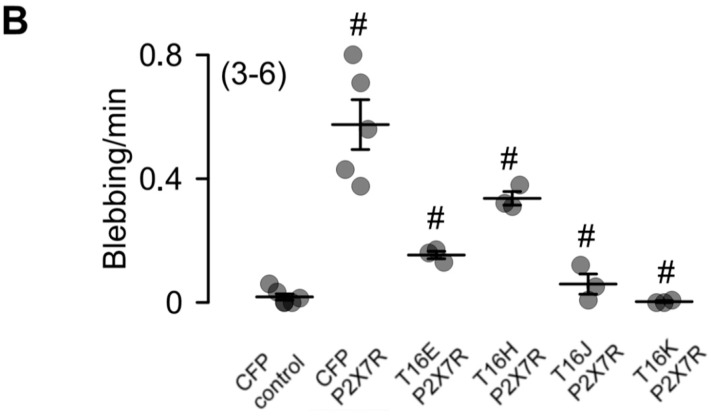
Reduced P2X7R-dependent membrane blebbing in cells expressing TMEM16 paralogs. (**A**) HEK293 cells were transfected with CFP (control), P2X7 receptor (P2X7R) and CFP or P2X7R and TMEM16 paralogs in a ratio of 4:1. Membrane blebbing was stimulated by 5 mM ATP. Blebbing events were counted in a time frame of 15 min after addition of ATP. Bar 20 µm. White arrows indicate blebbing events. (**B**) Summary of membrane blebbing rate (blebbing/min) of HEK293 cells expressing CFP (control), P2X7R and CFP or P2X7R and TMEM16 paralogs. Mean ± SEM (Number of experiments). For each experiment 10 CFP positive cells were measured), # significant difference when compared to control (ANOVA), α < 0.05.

Activation of P2X7 receptors (P2X7R) with 5 mM ATP for 15 min induced in P2X7R and CFP- transfected HEK293 cells 0.58 ± 0.08 blebbing events per min, but not in cells expressing CFP only (Figure 7A,B). Surprisingly, P2X7R-dependent membrane blebbing was significantly reduced in TMEM16E-, TMEM16J- and TMEM16K-CFP-CAAX-expressing cells, whereas TMEM16H-CFP-CAAX had no effect (Figure 7).

In summary, TMEM16E, TMEM16H, TMEM16J and TMEM16K proteins are typically located in intracellular compartments but can be targeted to the plasma membrane by addition of the CAAX motif to their C-terminus. At the plasma membrane, increased ion currents after purinergic stimulation or by increase in intracellular Ca^2+^ upon stimulation with ionomycin were inducible and were more pronounced in TMEM16E- and TMEM16K-CFP-CAAX and less distinct for TMEM16H- and TMEM16J-CFP-CAAX-expressing HEK cells. Surprisingly, expression of the TMEM16E, -J and -K paralogs at the plasma membrane did not induce phosphatidylserine scrambling and reduced P2X7R-dependent membrane blebbing. These results suggest that intracellular TMEM16 paralogs may have no impact on the lipid composition of intracellular membranes. Further experiments have to be performed to clarify the functional role of intracellular TMEM16 paralogs.

## 3. Discussion

A wide variety of molecules including nuclear lamins (intermediate filaments), Ras, GTP-binding proteins, several protein kinases and phosphatases contain specific amino acid sequences at their C-terminus. The so-called CAAX motif/box directs protein to the plasma membrane. C stands for cysteine residue, AA are two aliphatic residues and X represents any C-terminal amino acid, depending on different substrate specificities. These proteins always undergo a prenylation process before they are sent to plasma membrane [21,24]. Here, we showed that intracellular TMEM16 proteins containing a C-terminal CAAX box can be targeted to the plasma membrane (Figure 2C). This technique was also used to target the cAMP sensitive biosensor Epac2 to the plasma membrane. It was shown that Epac2 was targeted specifically to non-lipid raft domains of the plasma membrane [20].

We measured the anion conductance of the plasma membrane-targeted channels with two different methods: first, by YFP-quenching using ATP and the Ca^2+^ ionophore ionomycin, and second, by patch-clamp in whole-cell configuration after ionomycin application. To verify the YFP-quenching assay, we overexpressed TMEM16A-CFP as control and found increased basal iodide permeability, as reported earlier [4] (Figure 3A,D). Reducing the intracellular Ca^2+^ concentration with the Ca^2+^-chelator BAPTA attenuated basal activity of TMEM16A (Figure 3A,D). TMEM16A is activated through purinergic stimulation via P2Y receptors. It was shown that TMEM16A is organized in a functional compartment together with purinergic receptors and IP_3_ receptors of intracellular Ca^2+^ stores. Compartmentalization allowed for efficient purinergic Ca^2+^ signaling and activation of TMEM16A [5]. Such functional compartments were found in Xenopus oocytes and in dorsal root ganglia and were essential for efficient TMEM16A activation [25,26,27]. This compartmentalization by tethering the ER to the plasma membrane might allow for a restricted regulation of local Ca^2+^ concentrations. ER-tethering has also been described for the yeast TMEM16A-homolog Ist2 [28] and TMEM16H [11]. The increase in basal activity of TMEM16A (Figure 3A,D) could be explained by dislocation of overexpressed TMEM16A to non-lipid raft domains and activation by cytosolic Ca^2+^, which could be chelated by BAPTA (Figure 3A,D).

ATP induced iodide permeability (Ip) in HEK293 cells expressing TMEM16E-, TMEM16H- and TMEM16K-CAAX compared to CFP-expressing cells, which could not be further enhanced by ionomycin (Figure 3E–H). Similar results for TMEM16E-, TMEM16J- and TMEM16K-CAAX were obtained from the patch-clamp study (Figure 5). In contrast, activation of TMEM16H-CAAX could not be induced by ionomycin under whole-cell condition (Figure 5C). Tannic acid (20 µM), a non-specific blocker of TMEM16 paralogs, inhibited the ionomycin- or ATP-induced iodide permeability (Ip) as well as the ionomycin-induced whole-cell conductance, confirming activation of intracellular TMEM16 paralogs at the plasma membrane (Figure 4 and Figure 5).

TMEM16F is located at the plasma membrane and is an inducible Ca^2+^-activated lipid scramblase mediating passive transport of phospholipids, in particular phosphatidylserine (PtdSer), between both membrane leaflets [1,2,3]. The crystal structure of the fungal nhTMEM16 lipid scramblase indicated a lateral hydrophilic groove facing the plasma membrane, allowing for translocation of the hydrophilic head groups of phospholipids across the plasma membrane. This pathway is thought also to provide a nonspecific pathway for the anion permeability [29]. Other TMEM16 proteins, like TMEM16E, -H, -J and -K, are expressed mainly at the membranes of intracellular compartments [4,11,14,18,19] and are therefore difficult to study at the functional level. The first evidence that intracellular TMEM16 proteins operate as scramblases came from a chimeric approach in which a 35-aa-long stretch connecting transmembrane domains 4 and 5, designated “scrambling domains” in the TMEM16F protein [29], was introduced in the corresponding site of TMEM16A [30]. This study identified TMEM16E as a scramblase with a strong activity and TMEM16J and TMEM16K as scramblases with lower activities when compared to TMEM16F. For TMEM16H, no scramblase activity was found [30]. In our study, we could not confirm scrambling activity for TMEM16E, TMEM16J and TMEM16K (Figure 6). Moreover, the main parts of the cells which express the TMEM16-CAAX paralogs showed no annexin V staining after application of ionomycin (Figure 6A–D). To follow these surprising findings, we investigated P2X7 receptors (P2X7R)-dependent membrane blebbing, which is associated with PtdSer exposure [31,32].

P2X7R are ligand-gated, non-selective cation channels that are activated by high concentrations of extracellular nucleotides, released during inflammation, tissue injury and T-cell activation [33,34]. Activation of P2X7R by high extracellular ATP concentrations induced not only an instantaneous inward cationic current but also pore formation. This pore is non-selective and permeable to molecules up to 900 Da [31,32]. By the work of Ousingsawat et al., it was shown that activation of P2X7R caused Ca^2+^ influx, which activated—with a delay of seconds up to 2 min—a large whole-cell current and permeation of the fluorescent molecules YO-PRO-1, fluorescein and calcein [35,36,37,38]. Increase in intracellular Ca^2+^ by the P2X7R activated TMEM16F. TMEM16F was in part responsible for the delayed pore conductance and dye uptake [35]. TMEM16F also participated in many of the essential cellular responses downstream of P2X7R like initial cell shrinkage, membrane phospholipid scrambling, membrane blebbing and apoptosis [35]. Membrane blebbing was defined as a phenomenon in response to elevated intracellular Ca^2+^, where large membrane-bound vesicles (about 0.5 µm in diameter) protruded rapidly from the cell surface [39,40]. These vesicles were characterized by phosphatidylserines externalized to the outer membrane leaflet [31,32]. Blebbing has been observed during such processes as mitosis, cytokinesis, differentiation and apoptosis [40,41]. Targeting intracellular TMEM16 paralogs to the plasma membrane resulted in a reduction of P2X7R-dependent membrane blebbing induced by 5 mM ATP (Figure 7).

During apoptosis, platelet activation or formation of extracellular vesicles, a rapid externalization of PtdSer is occurring. It was found that the PtdSer concentration is 2- to 3-fold higher in lipid rafts as compared to the non-raft membrane [42,43,44,45]. Externalization of PtdSer to the outer leaflet in lipid rafts is dependent on TMEM16F activity [46,47,48,49]. Ionomycin or activation of P2X7R induces a strong increase in the intracellular calcium concentration, which activates endogenous TMEM16F [35]. Therefore, activation of TMEM16F caused PtdSer exposure and membrane blebbing in our experiments (Figure 6 and Figure 7). Overexpression of TMEM16-CAAX paralogs in the plasma membrane outside lipid rafts appears to counteract TMEM16F activity, possibly by scrambling PtdSer back to the inner leaflet and thus reducing PtdSer exposure. Such a scenario could explain the surprising finding that plasma membrane expression of intracellular TMEM16 paralogs inhibits ionomycin-induced scrambling and membrane blebbing.

In summary, the fusion of the C-terminal CAAX box moves intracellular TMEM16 proteins to the plasma membrane, which may be then assessable through functional studies. We showed that TMEM16E, -H, -J and -K produced Ca^2+^-activated anion currents, a result that corresponds well to our earlier findings [4]. Moreover, TMEM16E, -H, -J and -K reduced scrambling activity and P2X7R-dependent membrane blebbing. These results suggest that intracellular TMEM16 paralogs stabilize intracellular membranes by preventing scrambling activity and subsequent membrane blebbing. Further investigation is necessary to clarify the function of intracellular TMEM16 paralogs and to elucidate the mechanism of how these TMEM16 paralogs regulate membrane properties.

## 4. Materials and Methods

### 4.1. Cell Culture, cDNAs and Transfection

Human embryonic kidney HEK293T cells (ATCC, CRL-3216, LGC Standards GmbH, Wesel, Germany) and HEK293T stably expressing hsYFP-I152L (HEK-YFP, Amgen GmbH, München, Germany) were grown in DMEM low-glucose medium supplemented with 10% (*v*/*v*) FBS, 1% (*v*/*v*) l-glutamine and 10 mM HEPES (all from Capricorn Scientific, Ebsdorfergrund, Germany). HEK-YFP cells were cultured in the presence of selection antibiotic puromycin (0.5 µg/mL); Sigma-Aldrich, Missouri, USA). Fisher rat thyroid (FRT) cells were cultured in Invitrogen’s DMEM/F-12 GlutaMAX^TM^-I medium, supplemented with 5% fetal calf serum and 1% penicillin (100,000 units/L)/streptomycin (100 mg/L).

TMEM16A (NM_001378095.1), TMEM16E (NM_213599), TMEM16H (NM_020959), TMEM16J (NM_001012302) and TMEM16K (NM_001346468.1) cDNAs were cloned into the pcDNA3.1 plasmid. Cyan fluorescent protein (CFP) and the CAAX motif (KKKKSKTKCVIM) were cloned on the 3’-end of TMEM16 cDNA by standard cloning technique. CAAX motif was inserted by using the Primer 5´- ATATATTTAAATCTAGAAAAAAGAAGAAAAAGAAGTCAAAGACAAAGTGTGTA ATTATGTAAGGGCCCATATATTTAA-3´. T16A/T16E and T16A/T16K chimeras were generated by using the BEBuilder HiFi DNA Assembly cloning method (New England BioLabs, Frankfurt, Germany). All the primers were from Eurofins Genomics (Ebersberg, Germany). All constructs were verified by sequencing (SeqLab, Göttingen, Germany).

HEK293T cells were seeded in fibronectin- and collagen-coated 18 mm coverslips and transfected with plasmid vectors using standard protocols for Lipofectamine 3000 (Thermo Fisher Scientific, Germany). For cell blebbing experiments, HEK293T cells were transfected with human P2X7R (gift from Prof. Schmalzing, Institut für Pharmakologie & Toxikologie, Universitätsklinikum der RWTH Aachen) and CFP plasmids in the ratio of 4:1. All experiments were performed 48–72 h after transfection.

### 4.2. Immunocytochemistry

Transfected FRT cells were fixed for 10 min with 4%(*w*/*v*) paraformaldehyde as described previously [18]. In brief, cells were permeabilized and blocked with 2% (*w*/*v*, PBS) bovine serum albumin and 0.04% (*v*/*v*, PBS) Triton X-100. After 1 h incubation with primary antibody mouse anti-His tag (1:500, Qiagen, Hilden, Germany) at 37 °C, cells were incubated with a secondary donkey anti-mouse antibody conjugated with AlexaFluor 488 (1:1.000, Molecular Probes, Invitrogen). Nuclei were stained with Hoe33342 (0.1 µg/mL PBS, Aplichem, Darmstadt, Germany). β-Catenin (primary rabbit antibody from Sigma-Aldrich (C2206, Deisenhofen, Germany) was visualized using an Alexa 568-labeled secondary antibody. Cells were mounted on glass slides with fluorescence mounting medium (DAKOCytomation, Hamburg, Germany) and examined with an ApoTome Axiovert 200 M fluorescence microscope (Zeiss, Göttingen, Germany).

### 4.3. Annexin V Live Staining

TMEM16-CFP-CAAX transfected cells grown on glass coverslips were incubated with Annexin V Alexa Fluor 555 (0.9 µg/mL, Life Technologies, Germany) and Hoe33342 for 10 min at RT in Ringer solution (mmol/L: NaCl 145; KH_2_PO_4_ 0.4; K_2_HPO_4_ 1.6; Glucose 5; MgCl_2_ 1; Ca^2+^-Gluconate 1.3). To chelate intracellular Ca^2+^, cells were pre-incubated with 50 µM BAPTA-AM (Calbiochem; Merck, Germany) and 0.02% pluronic F-127 (Invitrogen, Thermo Fisher Scientific, Germany) for 30 min at RT. Live fluorescence images were obtained from a ApoTome 2 Axio Observer fluorescence microscope with ZEN software (Zeiss, Göttingen, Germany). CFP-fluorescence was monitored using the filter set #37 (excitation: 450/50 nm; emission: 510/50 nm), and for Annexin V Alexa Fluor 555 fluorescence the filter set #43 HE DS Red (excitation: 545/25 nm; emission: 605/70 nm) was used (Zeiss, Göttingen, Germany).

### 4.4. Iodide Quenching Assay

The iodide-sensitive enhanced yellow fluorescent protein (EYFP-I152L) was used to measure anion conductance as described previously [18]. Cells grown on coverslips were continuously perfused at 4–5 mL/min with Ringer solution (37 °C). YFP-I152L-fluorescence was excited at 485 nm and CFP-fluorescence at 440 nm to identify TMEM16-CFP-CAAX-positive cells (Figure S1) using a high-speed polychromatic illumination system for microscopic fluorescence measurements (Visitron Systems, Puchheim, Germany). The emitted light at 535 ± 25 nm was detected with a Coolsnap HQ CCD camera (Roper Scientific). Quenching of YFP-I152L-fluorescence by iodide influx was induced by replacing 20 mM extracellular chloride by iodide. Images were analyzed with Metafluor software (Universal Imaging). Changes in fluorescence induced by iodide were expressed as initial rates of fluorescence decrease (ΔF/Δt).

### 4.5. Whole-Cell Patch Clamp

Patch-clamp experiments in the fast whole-cell configuration were performed as described earlier [35]. In brief, cells were grown on coated glass coverslips, and these were mounted in a perfused bath chamber (37 °C) on the stage of an inverted microscope (IM35, Zeiss, Oberkochen, Germany). Patch pipettes were filled with a solution containing, in mM, CsCl 145, NaH_2_PO_4_ 1.2, Na_2_HPO_4_ 4.8, EGTA 1, Ca^2+^-Gluconate 0.209, d-Glucose 5, ATP 3; Ca^2+^ activity was 0.01 μM, pH 7.2. Patch pipettes had an input resistance of 2–4 MΩ. The access conductance was monitored continuously and was 60–140 nS. Currents and voltages were recorded using a patch-clamp amplifier (EPC 9, List Medical Electronics, Darmstadt, Germany). Data were analyzed using PULSE software (HEKA, Lambrecht, Germany). In regular intervals, membrane voltage (Vc) was clamped in steps of 20 mV from −100 to +100 mV from a holding voltage of −100 mV. Current density was calculated by dividing whole-cell currents by cell capacitance.

### 4.6. Materials and Statistic

All compounds used were of the highest available grade of purity. Data are reported as mean ± SEM. Student’s t-test was used for unpaired or paired samples comparison, and differences between more than two means were tested by using one-way analysis of variance (ANOVA) with a Bonferroni–Holm multiple comparisons test (post-hoc test), with significance assumed when *p* < 0.05.

## 5. Conclusions

Intracellular TMEM16 proteins can be targeted to the plasma membrane by adding a CAAX motif to their C-terminus and there they are assessable through functional studies. We could show that TMEM16E, -H, -J and K have inducible anion conductance and stabilize intracellular membranes by preventing scrambling activity and subsequent membrane blebbing. Further investigation is necessary to elucidate the mechanism of how these TMEM16 paralogs regulate membrane properties.

## Supplementary Materials

Supplementary materials can be found at https://www.mdpi.com/1422-0067/21/11/4065/s1, Figure S1: HEK293-YFP cells were transfected with (A) TMEM16A-CFP, (B) TMEM16E-CFP-CAAX, (C) TMEM16H-CFP-CAAX, (D) TMEM16J-CFP-CAAX and (E) TMEM16K-CFP-CAAX. An excitation wavelength of 485 nm was used to measure YFP-fluorescence, whereas an excitation wavelength of 440 nm was used to identify plasma membrane-targeted TMEM16E/-H/-J/-K-expressing cells by CFP-fluorescence. Fluorescence emission was measured at 520–540 nm. Bar 10 µm.

## Abbreviations

ANO	Anoctamin
ANO1	TMEM16A
ANO5	TMEM16E
ANO6	TMEM16F
ANO8	TMEM16H
ANO9	TMEM16J
ANO10	TMEM16K
P2X7R	P2X7 receptor
TMEM16	Transmembrane protein 16
