# Supplementary material for: Targeting of Intracellular TMEM16 Proteins to the Plasma Membrane and Activation by Purinergic Signaling
Source: Int J Mol Sci. 2020 Jun 5;21(11):4065. doi: 10.3390/ijms21114065 (PMC7312528; doi:10.3390/ijms21114065)
Supplement: Supplementary file 1 [file ijms-21-04065-s001.pdf]

Supplementary Materials

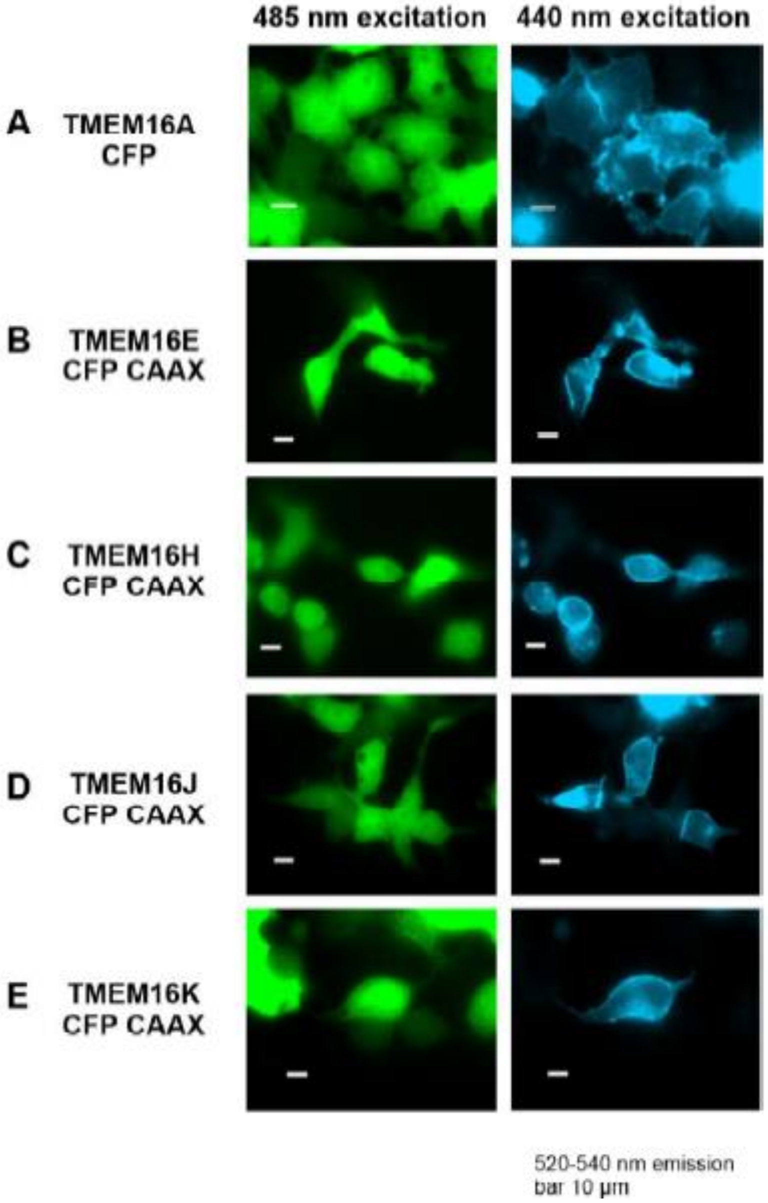

Figure S1. HEK293-YFP cells were transfected with (A) TMEM16A-CFP, (B) TMEM16E-CFP-CAAX, (C) TMEM16H-CFP-CAAX, (D) TMEM16J-CFP-CAAX and (E) TMEM16K-CFP-CAAX. An excitation wavelength of 485 nm was used to measure YFP-fluorescence, whereas an excitation wavelength of 440 nm was used to identify plasma membrane-targeted TMEM16E/H/J/K-expressing cells by CFP-fluorescence. Fluorescence emission was measured at 520–540 nm. Bar 10 µm.
